# Rolling pits of Hartmann’s mountain zebra (*Zebra equus hartmannae*) increase vegetation diversity and landscape heterogeneity in the Pre‐Namib

**DOI:** 10.1002/ece3.7983

**Published:** 2021-08-25

**Authors:** Thomas C. Wagner, Kenneth Uiseb, Christina Fischer

**Affiliations:** ^1^ Restoration Ecology School of Life Sciences Technische Universität München Freising Germany; ^2^ Directorate of Scientific Services Namibia Ministry of Environment, Forestry and Tourism Windhoek Namibia; ^3^ Faunistics and Wildlife Conservation Department of Agriculture, Ecotrophology, and Landscape Development Anhalt University of Applied Sciences Bernburg Germany

**Keywords:** bioturbation, ecosystem engineer, microsite, Namibia, savanna, trophic cascade, wallow

## Abstract

Microsites created by soil‐disturbing animals are important landscape elements in arid environments. In the Pre‐Namib, dust‐bathing behavior of the near‐endemic Hartmann's mountain zebra creates unique rolling pits that persist in the landscape. However, the ecohydrological characteristics and the effects of those microsites on the vegetation and on organisms of higher trophic levels are still unknown. In our study, we characterized the soil grain size composition and infiltration properties of rolling pits and reference sites and recorded vegetation and arthropod assemblages during the rainy season of five consecutive years with different amounts of seasonal rainfall. We further used the excess green vegetation index derived from drone imagery to demonstrate the different green up and wilting of pits and references after a rainfall event. In contrast to the surrounding grassland, rolling pits had finer soil with higher nutrient content, collected runoff, showed a higher infiltration, and kept soil moisture longer. Vegetation in the rolling pits was denser, dominated by annual forbs and remained green for longer periods. The denser vegetation resulted in a slightly higher activity density of herbivorous arthropods, which in turn increased the activity density of omnivorous and predatory arthropods. In times of drought, the rolling pits could act as safe sites and refuges for forbs and arthropods. With their rolling pits, Hartmann's mountain zebras act as ecosystem engineers, contributing to the diversity of forb communities and heterogeneity of the landscape in the Pre‐Namib.

## Graphical abstract

The dust‐bathing behavior of the Hartmann’s mountain zebra creates unique rolling‐pits that persist in the landscape. In contrast to the surrounding grassland, the rolling‐pits have finer soil with higher nutrient content, collect run‐off, show a higher infiltration and keep soil moisture longer. Vegetation in the rolling‐pits is denser, dominated by annual forbs and remains green for longer periods. This denser vegetation results in a slightly higher activity density of herbivorous arthropods, which in turn increases the activity density of omnivorous and predatory arthropods. In times of drought, the rolling‐pits act as safe‐sites and refuges for forbs and arthropods. With their rolling‐pits Hartmann’s mountain zebras act as ecosystem engineers, contributing to the diversity of forb communities and heterogeneity of the landscape in the Pre‐Namib. (Image credit: Theo Allofs, https://www.naturefineart.com).

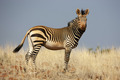

## 1 INTRODUCTION

Biopedturbation, the disturbance of soils by animals, is an important and often essential functional component of many ecosystems worldwide (Coggan et al., 2018). It determines the spatiotemporal characteristics of soil patches and thereby contributes to the ecosystems' diversity and heterogeneity (Mallen‐Cooper et al., 2019). Examples of the ecological importance of biopedturbation can be found in all climatic zones, but the focus is on arid regions (Coggan et al., 2018; Mallen‐Cooper et al., 2019). The spectrum of animals for these disturbances spans almost all animal groups (Coggan et al., 2018; Whitford & Kay, 1999) and even includes marine species such as the Californian Gray Whale (*Eschrichtius robustus*; Johnson et al., 1983) or the Antillean Manatee (*Trichechus manatus manatus*; Bacchus et al., 2009). The soil disturbances by animals affect both physical and chemical soil properties. These disturbances create vegetation‐free areas, shape soil topography, alter soil density and structure, change infiltration properties and soil moisture, influence the nutrient situation, and contribute to carbon cycling and nutrient turnover (for a detailed account of species and how they affect soil properties, see Coggan et al., 2018, Whitford & Kay, 1999 and Mallen‐Cooper et al., 2019). The large majority of documented soil disturbances by animals are due to burrows, mounds, or foraging digs, whereas reports about ground disturbances related to other behavior are relatively rare (Coggan et al., 2018; Whitford & Kay, 1999). Such described behavior‐related soil disturbances are all due to larger mammals and consequently result in relatively large structures; predominantly, depressions in the ground that serve as resting places are used for self‐grooming or even serve a social function. Examples of resting sites are the beds of the Nubian Ibex (*Capra nubiana*; Gutterman, 1997, 2001), the shelter sites of Gemsbok (*Oryx gazella*; Dean & Milton, 1991a, 1991b), or the hip holes of Kangaroos (*Macropus* spp.; Eldridge & Rath, 2002). As an example of resting depressions of a marine species, the resting holes of the Antillean Manatee (Bacchus et al., 2009) are known. Extensively studied and well‐documented are the wallows of the American Bison (*Bison bison*; McMillan et al., 2011, Nickell et al., 2018), which, in addition to rest and self‐grooming, also serve as a meeting place to promote social cohesion (Reinhardt, 1985). Also known are soil disturbances caused by the dust‐bathing behavior of many equids such as horses, donkeys (Moehlman, 1998), and zebras (Joubert, 1972; Skinner & Chimimba, 2005) that serves mainly for self‐grooming and the removal of ectoparasites.

The altered soil properties caused by the soil disturbance first directly affect the vegetation and via the trophic cascade eventually organisms of higher trophic levels (Coggan et al., 2018). The importance of such disturbances is especially high in arid environments. Here, with nutrient‐poor soils and the scarcity of water, the patterns and composition of vegetation are often linked to the geomorphological heterogeneity of the landscape (Bestelmeyer et al., 2006; Tongway & Ludwig, 2005). Small‐scale topographical features such as depressions and changes in soil properties form favorable microsites with higher nutrient and water availability (Bestelmeyer et al., 2006) that affect density and composition of the vegetation. The depressions created by animals collect runoff after rain and have better infiltration properties, increased soil moisture, and a higher nutrient content due to the accumulation of dung and urine (Mallen‐Cooper et al., 2019). The removal of existing vegetation further changes the competitive balance of plant communities (Mallen‐Cooper et al., 2019; Romero et al., 2015). Hence, such microsites offer more favorable conditions for plant recruitment and establishment than the surrounding matrix (Dean & Milton, 1991a, 1991b; Mallen‐Cooper et al., 2019).

The vegetation of such microsites is therefore often characterized by higher annual plant species richness, higher biomass production, and different plant community composition (Mallen‐Cooper et al., 2019). The more favorable conditions provided by these depressions are particularly important for annual forbs (Mallen‐Cooper et al., 2019; Whitford & Kay, 1999), which account for the largest share of species richness in arid grasslands, contribute to soil organic matter, and provide shelter and forage for arthropods, as well as larger animals and mammals (Siebert & Dreber, 2019). This in turn increases the activity density and diversity of arthropods (Nickell et al., 2018; Ruttan et al., 2016) or mammals (Ewacha et al., 2016). Such ecological effects can persist over many years or even decades (Coggan et al., 2018; Hastings et al., 2007; McMillan et al., 2011). Consequently, many soil‐disturbing animals act as ecosystem engineers and play an essential role in maintaining the diversity and productivity of the respective ecosystem (Mallen‐Cooper et al., 2019). Furthermore, such ecosystem engineers are often of particular concern for nature conservation (Hastings et al., 2007; Mullan Crain & Bertness, 2006), as their loss can lead to a deterioration in ecosystem function (Eldridge & James, 2009).

Coggan et al. (2018) list 121 species in their ecosystem engineering database, of which many occur in arid or dry regions. A species not yet included is the Hartmann's mountain zebra (*Equus zebra hartmannae*), a species that is endemic in the semidesert of Namibia's great escarpment, the Pre‐Namib, and a small part of Southern Angola and South Africa (Gosling et al., 2019). With their unique dust‐bathing behavior, the Hartmann's mountain zebras create characteristic small depressions, which even when abandoned remain in the landscape for many years (Skinner & Chimimba, 2005). None of the co‐occurring larger mammal species in the Namibian escarpment, Oryx (*Oryx gazella*), Greater Kudu (*Strepsiceros zambesiensis*), or Springbok (*Antidorcas marsuialis*), exhibits a similar behavior (Barandongo et al., 2018).

The exact effects of such rolling pits on the vegetation of arid savanna ecosystems and within the trophic cascade are largely unknown and have not been reported yet. With our study, we aim to elucidate the role of Hartmann's mountain zebras as ecosystem engineers in arid savanna ecosystems. Therefore, we investigated the effect of the rolling pits of Hartmann's mountain zebras over a period of five consecutive vegetation growing seasons with different amounts of rainfall on soil properties and density and composition of the vegetation. Further, we tested whether and how these soil and vegetation differences also affect ground‐dwelling arthropods.

## 2 METHODS

### 2.1 Study site and study species

**FIGURE A1:**
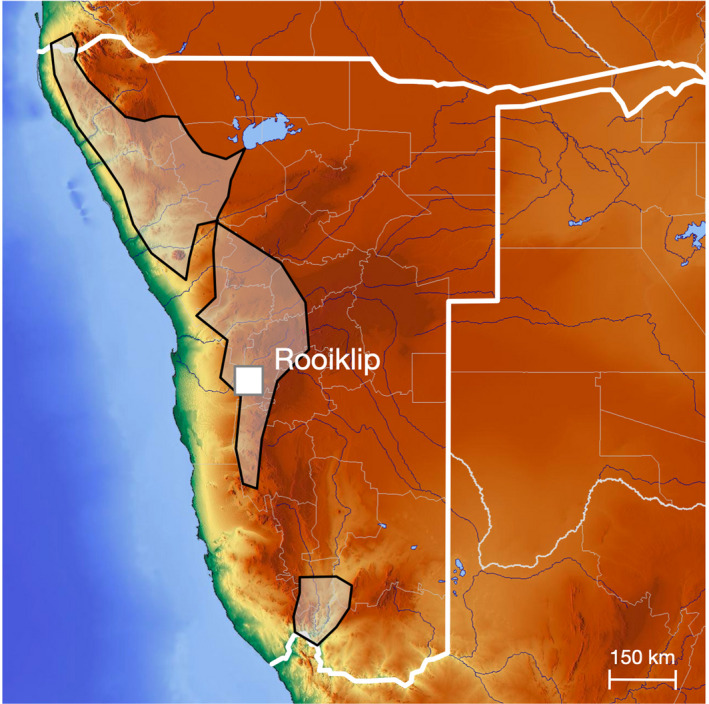
Distribution area of the Hartmann's mountain zebra (*Zebra equus hartmannae*) and location of the study site in Namibia. Position of the study site Rooiklip (S 23°24′23.29″, E 016°03′37.35″) and current distribution range of Hartmann's mountain zebra (transparent white area; www.iucnredlist.org, updated with data from the Environmental Information Service Namibia, Atlas of Mammals). The Hartmann's mountain zebra occurs in two disjunct areas within the Namibian escarpment, a smaller one in the southern part of Namibia, bordering South Africa, and a larger one, stretching from the Maltahöhe District north into the Iona National Park, southern Angola (Gosling et al., 2019)

Our study was conducted between 2014 and 2018 on the farm Rooiklip, Khomas, Namibia (33 K 612,448 7,411,832), situated at 1,000 m.a.s.l. in the center of the escarpment halfway between Windhoek and Walvis Bay (Figure A1). Namibia's great escarpment is an approximately 100‐km wide strip of rugged semidesert reaching from the border with South Africa in the South to Angola in the North. The climate is hot‐arid, mean annual precipitation ranges from ~50 mm in the West to ~200 mm at the upper edge in the East (Mendelson et al., 2009), but rainfall is highly variable and often erratic. At our study site, the mean annual precipitation is 120 mm, but ranged from 60 mm to 180 mm during the study period. Rainfall was measured on a daily basis with an on‐site standard rain gauge. The main vegetation growth period occurs from February to March, where two‐thirds of the annual precipitation occurs (Wagner et al., 2016). The vegetation is arid grassland dominated by perennial grasses of the genus *Stipagrostis* that is sparsely scattered with trees and occasional dwarf shrubs. Depending on the amount of rainfall, various annual forbs and annual grasses join these perennials. Total vegetation cover seldom reaches more than 15% (Wagner et al., 2016). Soils are leptosols and consist of a shallow layer of differently sized weathered particles of the underlying schist bedrock. Rooiklip and its neighboring farms are largely unfenced and allow wildlife to roam freely between the farms and from the edge of the escarpment to the Namib Desert.

The escarpment and the Pre‐Namib are home to the near‐endemic Hartmann's mountain zebra (Figure A1; Environmental Information Service, 2020; Gosling et al., 2019). The habitat of Hartmann's mountain zebra is mountainous areas and flats. Hartmann's mountain zebras further exhibit a marked migration pattern, following the rainfall and forage availability and moving between dry season and rainy season home ranges (Muntifering et al., 2019). Hartmann's mountain zebras usually live as family groups that consist of a stallion and his harem of mares and their foals. However, stallion groups or solitary stallions are frequent (Skinner & Chimimba, 2005), and occasionally, several family groups can join into herds with up to 30 individuals (Skinner & Chimimba, 2005).

Similar to many other equines, the Hartmann's mountain zebras show a regular dust‐bathing behavior (Joubert, 1972a), that in contrast to many other equines is carried out quite frequently several times during the day: The animal first moves into an incomplete lateral position and then turns alternately around its longitudinal axis to the left and right and then gives itself a push and stretches itself (McGreevy, 2012; Panzera et al., 2020). The exact reason for this behavior is unknown, but it is assumed that it is mainly for self‐grooming and removal of ectoparasites but partially also for resting (Joubert, 1972a). Typically, a family of Hartmann's mountain zebras prefers a very specific area for this procedure and often the individual spots are used by the group members, so that soon pronounced depressions in the soil are created that have on roughly 2.5 m diameter and can become up to 30 cm deep (Joubert, 1972b). When the zebras are migrating, the pits of the previous home range become abandoned but are sometimes reactivated during the next season, where they are used by the same or other families.

### 2.2 Microsite characteristics

In 2014, we mapped all detectable zebra pits within a 1 × 1 km area unused for livestock‐keeping since 2001. We searched the pits for signs of current use (tracks, fresh dung, lack of vegetation), noted the presence of zebra feces, and classified the pits as either active or abandoned. From all pits mapped in 2014, we randomly selected 16 abandoned pits, marked them, and paired them with a 2 m × 2 m reference site 5 m south of the center of the respective pit (Figure A4). We chose this particular size to match the average size of the rolling pits. The distance of 5 m was chosen to ensure similar topography and ground conditions.

We measured diameter and depth of the depression and determined the depth of the loose soil layer of the pit and reference sites using a simple penetrometer (Sutherland, 2006). We further characterized soil grain size composition of pits and reference sites by taking soil samples of the upper ten‐cm soil layer of a 40 × 40‐cm‐sized area. The samples were sieved into the different grain size classes: cobble and boulders, very coarse gravel, coarse gravel, fine‐to‐medium gravel, and sand and smaller particles, according to ISO 14688 (International Organization for Standardization, 2017), and the respective percentage by weight was calculated. Water‐soluble soil nutrients were determined by eluting an aliquot of 10 g the soil fraction sand and finer particles (oven‐dried at 105°C for 24 hr) with 100 ml of ultrapure water for 3 hr at room temperature. The eluate was left to sediment for 20 min, the supernatant was centrifuged for 15 min at 3,500 *g*, and 5 ml of the supernatant was used to analyze nitrogen and phosphate contents using standard ion chromatography (Michalski et al., 2019). A simulated precipitation event equivalent to 20 mm rainfall (applied within 1 hr) was used to measure the infiltration depth and determine the time course of soil moisture within the upper 10 cm of soil. Soil infiltration depth was determined 30 min after the precipitation event by digging and measuring the depth of the visually identified seepage front. The time course of soil moisture was determined using a TDR probe (Delta‐T Devices, ML3; Rajkai & Ryden, 1992) in two separate runs over 6 days each. The probe was initially calibrated once with an aliquot of soils taken from the pits and reference sites, respectively (Delta‐T Devices Ltd, 2013).

### 2.3 Vegetation assessment

From 2014 until 2018, we consecutively mapped the vegetation of pits and reference sites in two rounds at an interval of about 4 weeks during the respective vegetation period (Table A1). We determined the plant species present and their respective abundance and visually estimated the percentage of cover. Cover data were aggregated to the percentage of total plant cover, cover of annual forbs, and cover of annual grasses.

To show the development of vegetation over time and the differences in greening and wilting of pits and reference sites, we used high‐resolution RGB images acquired with a commercial DJI Phantom 4pro UAV. During the rainy season 2017, between 27 February and 6 April 2017, in total, 15 flyovers, 2 to 3 days between each other, were carried out over a transect covering the study area. The last relevant rain event occurred 14 days before the start of the flyovers and was 8 mm. During the flyovers, four distinct rainfall events occurred with 5 mm on 28 February, 22 mm on 2 March, 9 mm on 8 March, and 4 mm on 13 March. Flight altitude was 40 m.a.g.l., and image overlap was 75% to allow proper image alignment. All images of each flyover were processed and stitched as described in Woellner and Wagner (2019), resulting in a georeferenced (UTM, 33S) orthomosaic with a resolution of ~2 px/cm and a digital elevation model (DEM) of the study area.

### 2.4 Arthropod sampling

Coincident in time with each vegetation mapping, the occurrence, and abundance of ground‐dwelling arthropods were determined using pitfall traps. As the numbers of trapped arthropods depend not only on their abundance but also on their activity levels (e.g., Topping & Sunderland, 1992), the obtained numbers of arthropods are commonly referred to as “activity density” rather than abundance (Thiele, 1977). The resulting data were used to test for differences among arthropod assemblages between pits and references and to study bottom‐up effects along the trophic cascade.

Pitfall traps had a volume of 650 ml and a diameter of 9 cm and were filled with 250 ml of a 50% ethylene glycol—water mixture and a drop of detergent to reduce surface tension. Traps were installed on a level with the soil surface in the center of each pit or reference site (Lange et al., 2011) and kept open for 7 days after the vegetation was mapped. After each sampling round, arthropods were transferred into 70% ethanol and identified up to order and classified according to feeding guilds into herbivores, omnivores, and predators (Table A2; Picker et al., 2004; Scholtz & Holm, 2008) following the rapid assessment of biodiversity proposed by Oliver and Beattie (1996) used in comparable studies, for example, by Nickell et al. 2018 on Bison wallows. Though this somewhat limits deductions in the community structure, this was necessary as the majority of invertebrates have not been described (Samways & Samways, 1994). However, the method has been successfully applied to demonstrate habitat‐related changes in arthropod assemblages (see Blaum et al., 2009; Fabricius et al., 2003). Beetles (*Coleoptera*) and true bugs (*Hemiptera*) that included both species with herbivorous and predatory feeding preference were assigned to omnivores. Larvae that were not further determinable were excluded from analysis.

### 2.5 Statistics and data analysis

Statistical analysis was performed with R, version 3.6.3 (R Core Team, 2020). Comparisons of soil grain sizes and nutrients between pits and reference sites were done by permutational paired *t* tests (*perm.t.test*, library *RVAideMemoire*, Hervé, 2020). Time course of soil moisture was modeled as linear mixed effect model (*lme*; library *nlme* version 3.1‐120; Pinheiro et al., 2020). Soil moisture was log(x+1) transformed to obtain normality of variances. *Day* (since simulated rain event) and *Type* (pit/reference site) were used as explanatory variable.

Plant diversity was calculated as “effective number of species” (ENS), the equally common species that result in the respective Shannon–Wiener index and reflect the true diversity of the communities considered (Jost, 2006) using the function *diversity* from the *vegan* library (Oksanen et al., 2019). Indicator species, that is, plant species predominantly associated with either pits or reference sites, were identified using the *multipatt* function with *IndVal.g* as association function (library *indicspecies*; De Caceres & Legendre, 2009). The function provides estimates for the probability of a species occurring in the respective site group (*pO*) and a probability with which a site belongs to the respective group when the species is found (pT). It further calculates for each species an indicator value (*IndVal*) based on the product of its relative frequency and its relative average abundance in either pits or reference site.

Analysis of the drone data was done using the *raster* package (library *raster*, version 3.1‐5; Hijmans, 2020). Based on the orthomosaic, the “greenness” of each pixel was determined by using the *calc* function to calculate the excess green minus excess red vegetation index (ExGR; Meyer & Neto, 2008) that is suitable to differentiate vegetation from ground. The ExGR vegetation index is defined as 2*g‐r‐b – (1.4*r‐b), with r = R/(R+G+B), g = G/(R+G+B), and b = B/(R+G+B) and R, G, and B being the red, green, and blue values of the respective pixel. All zebra pits and the respective reference sites were digitized as polygons. The ExGR values within each polygon were sampled, and the mean was calculated with the *extract* function. A baseline value was established using the total mean of the ExGR values of all pits and reference sites at the beginning and end of the flyovers (days 0 and 38) when the vegetation was dry and wilted. In order to determine the slope at each zebra pit position, we used the *terrain* function to determine the slope of each cell of the DEM and aggregated the results to 5 m × 5 m cells applying the *aggregate* function with *mean*.

Differences between greenness of pits and reference sites were tested for each day using permutational *t* tests with *holm* adjustment for paired samples; differences between greenness of pits or reference sites to baseline were tested using standard permutational *t* tests (*perm.t.test*, library *RVAideMemoire*, Hervé, 2020) with *holm* adjustment.

To characterize the vegetation composition with regard to the prevalence of a certain functional group (annual grass, annual forb), we calculated the annual grass‐to‐forb ratio for each pit and reference site as the difference between the cover of annual grasses (annual grass cover/total cover) and proportional forbs cover (annual forb cover/total cover). This resulted in an index ranging between −1 for forbs only and +1 for grasses only. The relation between this grass‐to‐forb ratio and rainfall received within the preceding 30 days was modeled as linear mixed effect models (*lme*; library *nlme* version 3.1‐120; Pinheiro et al., 2020) using maximum likelihood with amount of rainfall (*Rain30d*), *Type*, and the two‐way interaction as explanatory variables.

For testing bottom‐up effects on arthropods, the activity density of the respective feeding group was modeled as linear mixed effect model (*lme*; library *nlme* version 3.1‐120; Pinheiro et al., 2020) using maximum likelihood. Models for herbivores included total vegetation cover and *Type* (pit/reference site) as explanatory variables, models for omnivores additionally contained the activity density of herbivores, and models for predators additionally contained the activity density of potential prey (herbivores and omnivores), as well as two‐way interactions of all explanatory variables. To obtain normality of errors, the response variables were log(x+1) transformed.

For all lmes, we used maximum likelihood and random intercept with pit/reference id as random factor to ensure independence of errors with respect to spatial and temporal autocorrelations (Pinheiro & Bates, 2000). Model simplifications were done in a backward stepwise model selection procedure by *stepAIC* (library MASS; Venables & Ripley, 2002) until the respective minimal adequate model was reached.

## 3 RESULTS

**FIGURE A3:**
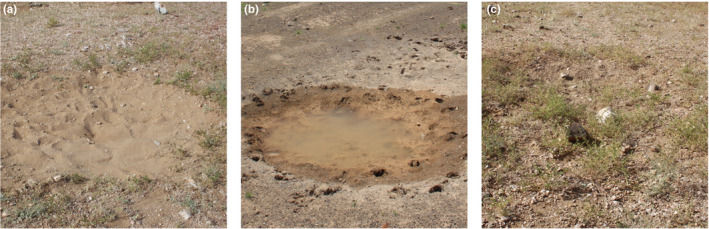
Zebra pits as microsites. Typical pits of Hartmann's mountain zebra. (a) active, with a pronounced ring of vegetation. (b) filled with runoff after rainfall. (c) abandoned and vegetated with various forbs

**FIGURE A4:**
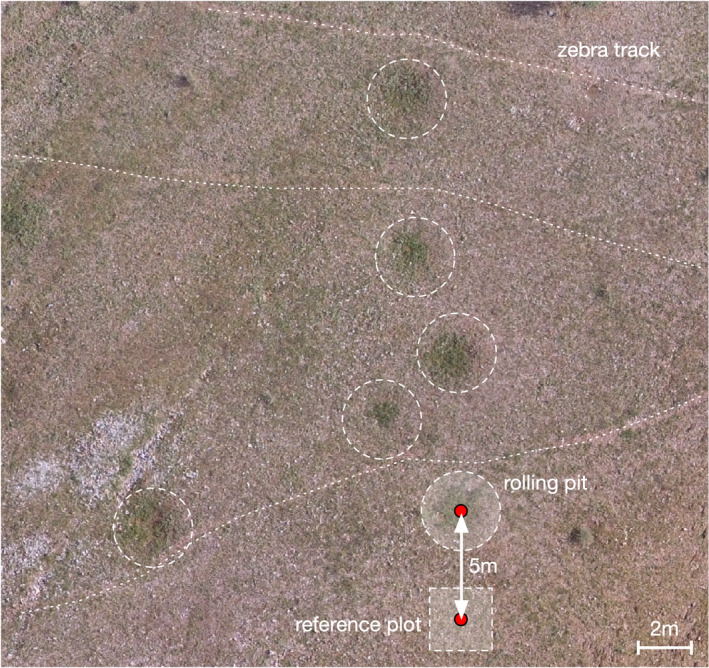
Aerial photograph with densely vegetated rolling pits. Details from an aerial photograph of the study site with zebra tracks (dotted lines) and six abandoned rolling pits (dashed circles) of mountain zebra 14 days after a rainfall event with ~30 mm of rainfall within 1 day. The dense annual vegetation clearly stands out against the less dense cover of the perennial *Stipagrostis* grasses of the matrix. The configuration of a research plot consisting of pit and reference site and the position of the pitfall traps (red dot) in the center are schematically illustrated

**FIGURE A5:**
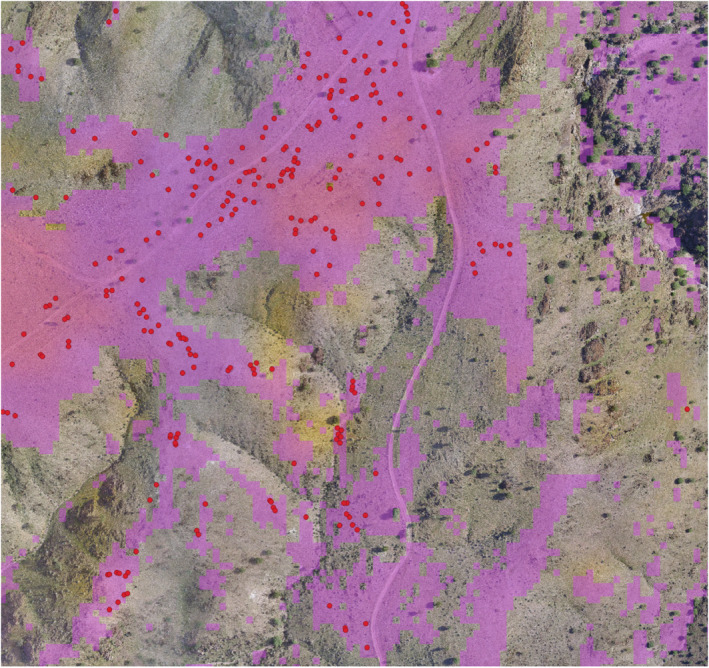
Position of the rolling pits within a section of the study site. Rolling pits only occur on level ground and often elevated grounds that make up about 1/3rd of the study site. Ground with an inclination of less than 5° is highlighted with magenta, red dots mark the respective pits

Between 2014 and 2018, in total, 656 pits were mapped in the study area, all restricted to elevated, but level and flat grounds, free of rocks, with an average inclination of <5° (Figure A5). From these 656 pits, 341 (~52%) were created in new places, where no pit has been mapped before, the rest was either re‐used or abandoned during the study period. Fresh or dried dung was found in 89% of the mapped pits. After heavy rainfall events, many rolling pits collected runoff and rainfall and it took up to three days for the water to evaporate (Figure A3b).

**FIGURE 1:**
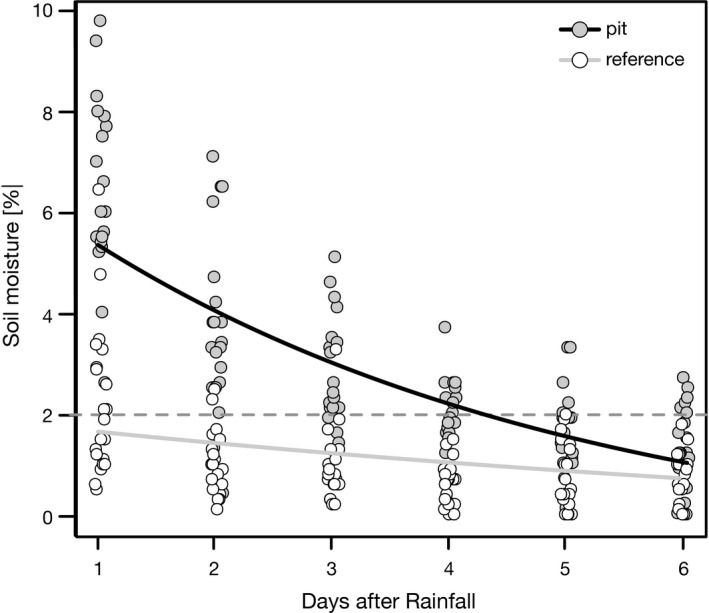
Trend of soil moisture in pits and reference sites after a simulated rainfall event with 20 mm applied within 1 h. Dashed horizontal line indicates permanent wilting point of sandy “soils”

Pit diameter ranged from 105 cm to 350 cm with a mean of 229 ± 55 cm (mean ± *SD*), resulting in an average area of 4.1 ± 0.3 m² per pit. The average depth of the pits (distance from the surface of the matrix to the soil layer of the pit) was 9.5 ± 3.6 cm. The substrate of pits contained considerably more fine‐grained material than the reference sites. In pits, sand and finer grained particles (84%) were the highest share, followed by gravel (11%), whereas reference sites contained 60% gravel and only 36% sand and finer material (Table A3). Water‐soluble soil nutrients were low (Table A4), with nitrogen and phosphate contents being significantly higher in pits (water‐soluble N: 4.8 mg/kg; PO_4_: 1.3 mg/kg) than on reference sites (water‐soluble N: 1.2 mg/kg; PO_4_: 0.5 mg/kg). The depth of the loose soil layer of pits was 29.3 ± 8.1 cm and thereby significantly higher than the average loose soil depth 5.5 ± 2.1 cm of the reference sites (*p* < .002). After our simulated rain event, the soil infiltration depth in pits reached 9.9 ± 1.9 cm and only 4.7 ± 1.7 cm on reference sites, which corresponded with the depth of loose soil. During the first 24 hr after the event, the average soil moisture in pits reached 6.31 ± 0.24% and remained above the permanent wilting point for coarse sand (~2%) for over four days. In contrast, soil moisture never exceeded the permanent wilting point on reference sites (Figure 1).

**FIGURE 2:**
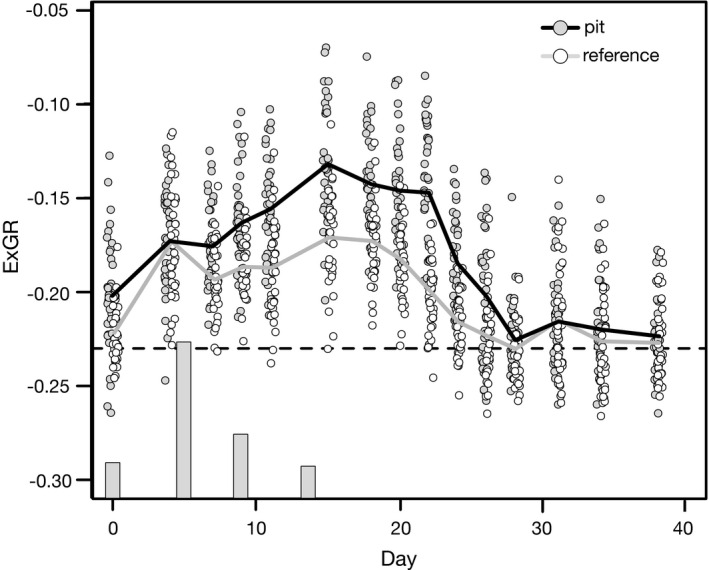
Greenness of pits and reference sites expressed by the ExGR vegetation index, derived from RGB UAV imagery taken on March 2017. Dashed line represents baseline of soil without green vegetation. Gray bars indicate rainfall events with 5, 22, 9, and 5 mm

The temporal course of greening and drying out of the vegetation following natural rain events showed clear differences between pits and reference sites. The ExGR vegetation index and hence greenness of the zebra pits increased with each rainfall event until day 14 and then decreased steadily over the next 14 days until it reached the baseline at day 28 when the vegetation was wilted. From day six on, it was always above the greenness of the reference sites. In contrast, the greenness of the reference sites increased only after the first rain and then remained constant, apart from a slight decrease on days six and eight, until day 18. After that, it decreases and reaches the baseline around day 26, about two days earlier than the pits (Figure 2; Table A5).

**FIGURE 3:**
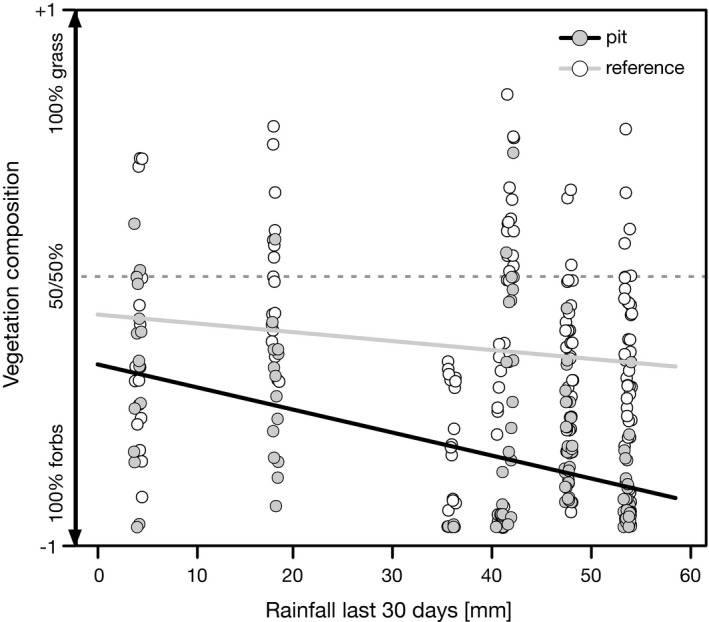
Relative proportion of annual forbs and grasses in relation to rainfall in the preceding 30 days in pits and reference sites. Dashed line indicates equal grass‐to‐forb ratio

Total vegetation cover did not exceed 23% in pits and 13% on reference sites and correlated with rainfall received within the preceding 30 days. Both total vegetation cover and cover of annual forbs were higher (total cover on average 1.4‐fold, cover of annuals 1.8‐fold) in pits compared to reference sites throughout all years (Table A6). The proportion of forbs and the total cover of annual plants was significantly higher in pits and increased significantly with the amount of rainfall received during the preceding 30 days (Figure 3; Table A6).

In total, we found 26 annual plant species, from which six were annual grasses and 20 annual forbs, but all native to the area. General plant diversity of pits and reference sites was similar with 4.8–8.4 species in pits and 4.5–7.1 species in reference sites (Table A6) but varied throughout the years as a response to different amounts of rainfall. Pits were mainly characterized by the presence of annual forbs (seven indicator species), whereas annual grasses mainly characterized the reference sites (three indicator species; Table 1).

**TABLE 1:** Species associated predominantly with pits or reference sites as identified by indicator analysis

	Growth form	pT	pO	IndVal	*p*‐value
Rolling pits					
*Heliotropium ciliatum*	Annual forb	0.89	0.26	0.527	.001
*Hirpicum gazanoides*	Annual forb	0.84	0.27	0.511	.001
*Sesbania pachycarpa*	Annual forb	0.97	0.22	0.443	.001
*Cucumis sagittatus*	Annual forb	1.00	0.16	0.432	.001
*Hermannia modesta*	Annual forb	0.83	0.06	0.248	.025
*Cleome suffruticosa*	Annual forb	0.80	0.05	0.204	.052
*Citrullus lanatus*	Annual forb	1.00	0.01	0.122	.513
Reference sites					
*Enneapogon desvauxii*	Annual grass	0.84	0.79	0.843	.001
*Eragrostis nindensis*	Annual grass	0.96	0.15	0.377	.001
*Entoplocamia aristulata*	Annual grass	0.75	0.15	0.367	.007

Note

IndVal, indicator value, product of the relative frequency and relative average abundance in pits or reference sites (De Caceres & Legendre, 2009); pO, probability of finding the species within group; pT, estimate of probability that site belongs to target group when species has been found.

Herbivorous arthropods showed a significant positive correlation with total vegetation cover with slightly higher activity density in pits compared to reference sites. Omnivores were positively related to the activity density of herbivores, but without differences between pits and reference sites, whereas predators were showing a significant positive correlation with the activity density of prey (omnivores and herbivores) but no significant difference between pits and reference sites (Table 2).

**TABLE 2:** Coefficients of the relationship between the activity density of the different arthropod groups and the respective explanatory variables determined with the mixed effect models

Explanatory variable	Activity density herbivores	Activity density omnivores	Activity density predators
Vegetation cover	1.02***	–	–
Herbivores	n.t.	1.04***	1.03***
Omnivores	n.t.	n.t.	1.02***
Type^a^	0.84*	–	–

Note

All interactions were excluded during stepwise simplification. Back‐transformed, exponentiated coefficients, and levels of significance are given. “n.t.”: not tested. “Herbivores” include the orders *Caelifera*, *Cicadina*, and *Stenorrhyncha*; “omnivores” include *Blattodea*, *Coleoptera*, *Ensifera*, *Heteroptera*, and *Psocoptera*; “predators” include *Acarina*, *Araneae*, *Chilopoda*, *Pseudoscorpiones*, and *Scorpiones*.

*p < .05, **p < .01, ***p < .001.

^a^ “pit” versus “reference site”; reference level was “reference site.”

## 4 DISCUSSION

The characteristic rolling pits of the Hartmann's mountain zebra in the Pre‐Namib, created by their dust‐bathing behavior, produce favorable microsites that clearly differ from their surroundings. Their special ecohydrological properties promote a denser and different vegetation composition, characterized by annual forbs, and, to a lesser extent, increase the activity density of herbivorous arthropods. In the escarpment region of Namibia, no other larger mammal species exhibits a comparable rolling or digging behavior leading to characteristic depressions similar to the rolling pits of the Hartmann's mountain zebra (Barandongo et al., 2018).

With an average diameter of 230 cm and a total depth of up to over 40 cm, the rolling pits of the Hartmann's mountain zebra are within the larger discrete structures created by soil‐disturbing animals, although they did not reach the size of bison wallows (Miller et al., 2013) that cover in average almost twice the area. The rolling pits are filled up to ¾ with a 30‐cm thick layer of predominantly loose sand. By their dust‐bathing behavior, the mountain zebras remove the extant vegetation dominated by perennial grasses of the genus *Stipagrostis* and coarser soil components such as gravel and cobble are moved outside of the pit, leaving the finer material in the pits themselves.

The characteristic of the rolling pits generally corresponds to those known from depressions and resting places of larger antelopes, equines, or kangaroos. Similar to the bedding sites of the Nubian ibex (Gutterman, 1997) or the wallows of American bison (McMillan et al., 2011), the rolling pits of Hartmann's mountain zebra collect runoff after rainfall. The loose soil filling of the pits allows a better infiltration, and once infiltrated, this moist soil acts as a storage for the runoff water that keeps the soil moist for a few days longer than the surrounding soil. In addition, the loose soil of rolling pits has a higher water‐soluble nitrogen and phosphate contents. Nine of ten pits we mapped had considerable remains of dry zebra dung. Deposits of feces and higher urine concentrations in the soil are a typical feature known from other resting places of larger animals such as Gemsbok (Dean & Milton, 1991a, 1991b) or Kangaroo (Eldridge & Rath, 2002). In addition, such depressions frequently accumulate litter (Mallen‐Cooper et al., 2019), which is then together with the dung quickly converted into nutrients by the stronger mineralization due to a higher soil moisture (Eldridge & Rath, 2002; Veldhuis et al., 2018). As a result, these resting places and so the rolling pits of the Hartmann's mountain zebra form relatively moist and nutrient‐rich patches in the otherwise nutrient‐poor savanna (Augustine & McNaughton, 2006; McNaughton et al., 1988).

The pits are mainly used for only one season but remain as depressions for several years. When the vegetation dries up, the Hartmann's mountain zebras move to their dry season home ranges (Muntifering et al., 2019) and the pits become abandoned. In the following rainy season, when the zebras return, new pits are created. From 656 abandoned pits, between 2014 and 2018, only 71 (11%) were reactivated, while at the same time, 341 were newly created. When abandoned, the higher nutrient availability and an increased and longer lasting soil moisture allow the establishment of vegetation that is clearly denser and differs in terms of vegetation composition from the surrounding grassland similar to the effects of the abandoned wallows of the American bison or the bedding sites of the Nubian ibex (Gutterman, 1997; Nickell et al., 2018).

Pits and reference sites show a simultaneous green flush, but the greenness of pits soon exceeds that of the reference sites, a clear sign of denser vegetation within the pits. The greenness of pits remains higher until it reaches the baseline of dry ground about two to four days later than the reference sites. This delayed wilting corresponds with the finding of our infiltration experiments, where soil moisture in pits remained over the permanent wilting point for two days longer. Ground truthing and our assessment of the vegetation in the field confirms a higher cover and a different structure of the vegetation of the pits. Though the general plant diversity of pits and reference sites is similar, the vegetation of pits and reference sites differs in terms of species composition and dominant functional types. The typical tufted perennial grasses of the genus *Stipagrostis* that make up the dominant vegetation of the surrounding grassland are practically absent in the pits. Instead, the pits are mainly vegetated with annuals, with a clear dominance of forbs. This dominance of forbs becomes even more pronounced with higher rainfall, when sufficient runoff can be collected and stored by the soil in the pits. The general importance of animal‐created depressions in arid environments for annual forbs has been documented for a number of animal species (Coggan et al., 2018; Gutterman, 2001). The higher and longer lasting soil moisture of the rolling pits also plays a crucial role in the occurrence of annual forbs. Together with higher nutrient availability, it is a decisive factor for the establishment of forbs in arid environments (Siebert & Dreber, 2019), which need longer lasting favorable conditions for the successful establishment of their seedlings (Hillel & Kozlovsky, 2012). The lack of perennial grasses in the pits further ensures that freshly germinated ephemerals are subject to less competition (Dean & Milton, 1991a, 1991b). Many of the annual forbs found in the pits such as *H. ciliatum*, *H. modesta*, or *S. pachycarpa* are otherwise mainly found at sites with better water supply, such as dry riverbeds or topographic depressions (personal observation, Strohbach, 2017).

Annual forbs represent the main component of species diversity in savanna ecosystems. Annual forbs contribute substantially to various ecosystem functions and provide forage and shelter for numerous arthropod species (Siebert & Dreber, 2019). Given the generally sparse vegetation and the particularly low density of forbs in the study region, zebra pits are likely to be an important factor shaping the local forb communities and providing safe sites for forbs particularly under dry conditions. Therefore, pits may act as stepping stones, linking the vegetation of the intermittent streams and allowing forb species to spread not only along these rivers but also via the surrounding landscape.

Similar to Nickell et al. (2018) or Ewacha et al. (2016), we found that the effects of pits were not restricted to vegetation but also affected higher trophic levels and slightly increased the activity density of arthropods. Independent of vegetation cover, herbivorous arthropods were generally more abundant in the pits. The different and denser vegetation of the abandoned pits provides a good source of nutrition for herbivores (Dalerum et al., 2017) and the larvae of the other feeding guilds. The structural diversity and conditions of the herbaceous vegetation further offer a variety of niches and provide habitats and refuges for arthropod species and their larvae (Dalerum et al., 2017). However, the size of these effects on arthropod activity density is not very pronounced, which is probably due to the limited size of the rolling pits and arthropod mobility, as well as the low resolution of the determination of the different arthropod taxa.

Even though our study was only carried out on a single farm and an area comprising only 1 km^2^, our results can be transferred to the entire geographic region. Flyovers with UAV and observations on other farms in the region confirm the more or less dense occurrence of rolling pits within larger areas. Climate, precipitation, and vegetation in the distribution range of Hartmann's mountain zebra are largely similar (see Mendelson et al., 2009). The rolling pits persist for many years after they have been abandoned and thus most likely have a long‐lasting influence on the ecosystem. The current number of mountain zebras is estimated to be 32.000 individuals (Gosling et al., 2019). Their seasonally shifting home ranges (Muntifering et al., 2019), the long persistence, and limited reuse of abandoned pits result in an occurrence of mountain zebra pits throughout the Namibian escarpment in a varying density (Wagner TC & Fischer C, unpublished data). In conclusion, with their rolling pits, the Hartmann's mountain zebras clearly contribute to a higher biodiversity and structural heterogeneity of the arid savanna landscape in the Pre‐Namib and can therefore be, like other soil‐disturbing animals (Mallen‐Cooper et al., 2019; Romero et al., 2015), considered as an ecosystem engineer. In the future, the importance of the rolling pits for the local forb and arthropod communities might even increase, due to prolonged severe drought periods, which are expected in the region as a result of climate change (Archer et al., 2019; Maúre et al., 2018).

## CONFLICT OF INTEREST

The corresponding author confirms on behalf of all authors that there have been no involvements that might raise the question of bias in the work reported or in the conclusions, implications, or opinions stated.

## AUTHOR CONTRIBUTIONS

**Thomas C Wagner:** Conceptualization (equal); Formal analysis (equal); Investigation (equal); Methodology (equal); Supervision (equal); Writing‐original draft (equal). **Kenneth Uiseb:** Formal analysis (equal); Investigation (equal); Writing‐original draft (equal); Writing‐review & editing (equal). **Christina Fischer:** Conceptualization (equal); Formal analysis (equal); Investigation (equal); Methodology (equal); Supervision (equal); Writing‐original draft (equal); Writing‐review & editing (equal).

## Figures and Tables

**FIGURE A2:**
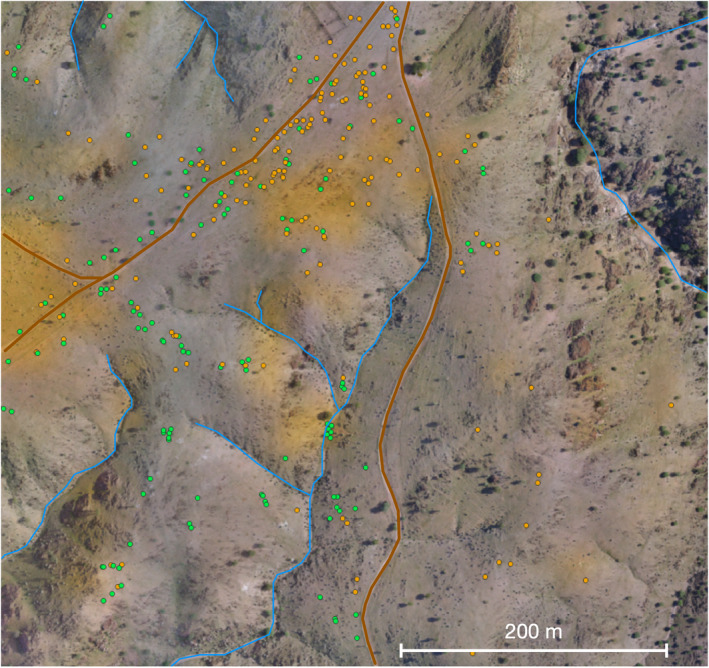
Distribution of zebra pits. Distribution of active (green) and abandoned (orange) zebra pits within a 500 m × 500 m section of the study site in 2017. The pits are clustered on level, open areas between intermittent rivers (blue lines) and ravines and along tracks (brown lines)

## Data Availability

Data are available in the OSF Data repository under https://doi.org/10.17605/OSF.IO/QWX6K.

## 1

**TABLE A1:** Vegetation mapping and arthropod sampling dates

Year	Run	Date of vegetation sampling and opening of pitfall traps	Rainfall within the preceding 30 days [mm]	Remark
2014	1	10.03.2014	0	No vegetation
	2	22.03.2014	54	
2015	1	07.03.2015	4	
	2	08.04.2015	18	
2016	1	12.03.2016	48	
	2	23.03.2016	48	
2017	1	11.03.2017	36	
	2	30.03.2017	41	
2018	1	20.03.2018	0	No vegetation
	2	24.04.2018	42	

Note

Dates when vegetation sampling and pitfall trapping took place and the total amount of rainfall received in the preceding 30 days. The remark “no vegetation” indicates that no vegetation was found.

**TABLE A2:** Arthropod groups

Group	Included taxonomic units
Herbivores	*Caelifera* (Short‐horned grasshoppers) *Cicadina* (Cicadas) *Stenorrhyncha* (Aphids and scale insects)
Omnivores	*Blattodea* (Cockroaches) *Coleoptera* (Beetles) *Ensifera* (Long‐horned grasshoppers) *Heteroptera* (True bugs) *Psocoptera* (Psocids and book lice)
Predators	*Acarina* (Ticks and mites) *Araneae* (Spiders) *Chilopoda* (Centipedes) *Pseudoscorpiones* (False scorpions) *Scorpiones* (Scorpions) *Solifugae* (Solifugae)

Note

Classification of arthropod orders according to their feeding preference. Arthropods were determined to order and grouped according to their feeding behavior using Picker et al., 2004; Scholtz & Holm, 2008; http://biodiversity.org.na/taxondisplay.php?nr=8, downloaded: 04.07.2017. Orders with both herbivorous and predatory representatives were classified as omnivores.

**TABLE A3:** Soil grain size composition and chemistry

Grain size according to EN/ISO 14688‐1:2002	Reference (mean ± *SD*) [%]	Pit (mean ± *SD*) [%]	*p*‐value
Sand and finer	35.5 ± 15.5	84.3 ± 10.7	.001 **
Fine to medium gravel	30.1 ± 11.6	11.7 ± 4.7	.004 *
Coarse gravel	30.3 ± 8.4	1.1 ± 0.8	.001 **
Very coarse gravel	3.4 ± 3.4	2.3 ± 3.4	.400 n.s.
Cobble	0.6 ± 1.3	0.6 ± 0.7	.944 n.s.
Boulders	0.1 ± 0.2	0.0 ± 0.1	.000 n.s.

Note

Soil sample from the upper 10 cm of a 40 × 40‐cm‐sized plot of pits and references, taken after the end of the study. Percentage by weight of the different grain size classes (ISO 14,688). Significance of difference determined with paired permutational *t* tests (*perm.t.test*, *RVAideMemoire*; *n* = 999); ^*^ *p* < .05, ^**^ *p* < .01, ^***^ *p* < .001; n.s: not significant.

**TABLE A4:** Water‐soluble soil nutrients

Parameter	Reference (mean ± *SD*) [mg/kg]	Pit (mean ± *SD*) [mg/kg]	Significance of difference
pH	7.41 ± 0.27	7.52 ± 0.28	0.180 n.s.
N	1.22 ± 0.07	4.80 ± 3.46	0.012 **
PO4	0.48 ± 0.28	1.33 ± 0.78	0.012 **
Na	0.22 ± 0.08	0.45 ± 0.15	0.631 n.s.
K	6.76 ± 5.00	6.63 ± 5.31	0.924 n.s.
Mg	3.02 ± 2.27	5.78 ± 4.78	0.250 n.s.
Ca	6.84 ± 3.91	8.77 ± 1.82	0.344 n.s.

Note

Aliquot of 10 g oven‐dried substrate (sand and finer) eluted with 100ml of ultrapure water for 3h at room temperature; eluate left to sediment for 20min, supernatant centrifuged for 15min at 3,500 g; 5m of supernatant analyzed using standard ion chromatography. Significance of difference between pits and reference determined with paired permutational *t* tests (*perm.t.test*, *RVAideMemoire*; *n* = 999); ^*^ *p* < .05, ^**^ *p* < .01, ^***^ *p* < .001; n.s: not significant.

**TABLE A5:** Greenness

Day	ExGR		Significance		
	Pit	Reference	Pit–Reference	Pit‐Baseline	Reference‐Baseline
0	−0.208 ± 0.030	−0.221 ± 0.016	***	***	*
4	−0.170 ± 0.028	−0.172 ± 0.029	n.s.	***	***
7	−0.173 ± 0.023	−0.193 ± 0.019	***	***	***
9	−0.161 ± 0.025	−0.186 ± 0.019	***	***	***
11	−0.153 ± 0.028	−0.186 ± 0.024	***	***	***
15	−0.129 ± 0.031	−0.170 ± 0.024	***	***	***
18	−0.140 ± 0.027	−0.172 ± 0.021	***	***	***
20	−0.145 ± 0.031	−0.182 ± 0.020	***	***	***
22	−0.141 ± 0.043	−0.198 ± 0.018	***	***	***
24	−0.184 ± 0.026	−0.215 ± 0.016	***	***	*
26	−0.196 ± 0.050	−0.223 ± 0.021	***	***	n.s.
28	−0.224 ± 0.021	−0.230 ± 0.017	n.s.	n.s.	n.s.
31	−0.214 ± 0.026	−0.214 ± 0.031	n.s.	*	n.s.
34	−0.219 ± 0.027	−0.223 ± 0.026	*	n.s.	n.s.
38	−0.223 ± 0.024	−0.224 ± 0.021	n.s.	n.s.	n.s.

Note

n.s: not significant, ****p* < .001; ***p* < .01, **p* < .05. Average ExGR values and standard deviation of pits and references during the flyovers and the significance of difference between pits and references and the respective difference to baseline.

**TABLE A6:** Total plant cover, cover of annual forbs and grasses and effective number of species (mean ± *SD*) in pits and on references

Param.	2014		2015		2016		2017		2018	
Rain 30 d [mm]	52		18		48		41		36	
	Pit	Ref.	Pit	Ref.	Pit	Ref.	Pit	Ref.	Pit	Ref.
Total plant cover [%]	6.7 ± 3.9	6.6 ± 5.5	5.0 ± 4.9	3.7 ± 2.0	18.3 ± 13.9	13.1 ± 8.3	22.9 ± 14.0	11.7 ± 6.7	0.3 ± 0.3	0.2 ± 0.2
Annual forb cover [%]	6.5 ± 3.9	3.9 ± 2.6	3.9 ± 0.4	2.9 ± 2.0	16.7 ± 12.5	10.1 ± 8.1	22.8 ± 14.0	8.1 ± 6.4	0.3 ± 0.3	0.2 ± 0.2
Annual grass cover [%]	0.19 ± 0.28	0.96 ± 0.60	0.65 ± 0.53	1.19 ± 0.84	1.67 ± 1.29	2.42 ± 1.69	0.03 ± 0.09	0.10 ± 0.15	0.07 ± 0.14	0.19 ± 0.26
Species	8.4 ± 2.7	6.7 ± 1.7	6.2 ± 2.2	7.1 ± 2.2	5.3 ± 1.5	5.6 ± 1.7	6.8 ± 1.7	7.1 ± 1.7	4.9 ± 1.3	4.5 ± 2.1
ENS	5.8 ± 2.3	3.8 ± 1.4	3.1 ± 1.9	3.5 ± 1.5	3.0 ± 1.3	2.8 ± 1.3	4.6 ± 1.5	5.3 ± 1.5	3.0 ± 1.2	2.2 ± 1.0

Note

Plant diversity of pits and references as average number of species and effective number of species (ENS, Jost, 2006; Tuomisto, 2010). No relevant vegetation was found in 2019.
